# The severity of experimental arthritis is independent of IL-36 receptor signaling

**DOI:** 10.1186/ar4192

**Published:** 2013-03-01

**Authors:** Céline Lamacchia, Gaby Palmer, Emiliana Rodriguez, Praxedis Martin, Solenne Vigne, Christian A Seemayer, Dominique Talabot-Ayer, Jennifer E Towne, Cem Gabay

**Affiliations:** 1 Division of Rheumatology, Department of Internal Medicine, University Hospital of Geneva, 26 avenue Beau-Séjour, 1211 Geneva 14, Switzerland; 2 Department of Pathology-Immunology, University of Geneva School of Medicine, 1 rue Michel-Servet, 1211 Geneva 4, Switzerland; 3 Novartis Pharma AG, Translational Medicine, NIBR, WSJ386.10.48, PO Box, 4002 Basel, Switzerland; 4 Department of Inflammation Research, Amgen Inc., 1201 Amgen Court West, Seattle, WA 98119, USA

## Abstract

**Introduction:**

Interleukin (IL)-36 refers to three related IL-1 family cytokines, IL-36α, IL-36β, and IL-36γ, that bind to the IL-36 receptor (IL-36R). IL-36 exerts proinflammatory effects in skin and lung and stimulates T cell responses. In the present study, we examined the expression and function of IL-36R and its ligands in experimental arthritis.

**Methods:**

Collagen-induced arthritis (CIA), antigen-induced arthritis (AIA), and K/BxN serum transfer-induced arthritis were induced according to standard protocols. Messenger RNA levels for IL-36R and its ligands in the joints of mice with CIA were determined by RT-qPCR. Mice with CIA were injected with a blocking monoclonal anti-IL-36R, a blocking anti-IL-1RI, or their isotype-matched control antibodies at the time of arthritis onset. Anti-IL-36R or control antibodies were also injected at the time of AIA induction. Finally, IL-36R-deficient mice were examined in AIA and serum transfer-induced arthritis. The development and severity of arthritis were assessed by clinical and histological scoring.

**Results:**

IL-36R, IL-36Ra and IL-36γ mRNA were detected in the joints of mice with CIA, but their levels did not correlate with arthritis severity. As opposed to anti-IL-1RI antibody treatment, the injection of an anti-IL-36R antibody was devoid of effect on the development and severity of CIA. The severity of joint inflammation and structural damage in AIA was also unaltered by anti-IL-36R antibody treatment. Finally, the severity of AIA and K/BxN serum transfer-induced arthritis was similar in IL-36R-deficient and wild-type mice.

**Conclusions:**

The development and severity of experimental arthritis are independent of IL-36R signaling.

## Introduction

The IL-1 family of cytokines includes three well-described agonists with pro-inflammatory properties, namely IL-1α, IL-1β, and IL-18, as well as the IL-1 receptor antagonist (IL-1Ra), a naturally occurring inhibitor that regulates the biological activities of IL-1α and IL-1β. In addition, seven novel IL-1 family members have been identified on the basis of their sequence homology, three-dimensional protein structure, gene location and receptor binding profile [1-7]. These proteins are now termed IL-36Ra, IL-36α, IL-36β, IL-36γ, IL-37, IL-38 and IL-33 (previously known as IL-1F5, IL-1F6, IL-1F8, IL-1F9, IL-1F7, IL-1F10 and IL-1F11, respectively) [8]. IL-36α, IL-36β and IL-36γ bind to a heterodimeric receptor consisting of the IL-36 receptor (IL-36R) subunit (previously called IL-1Rrp2) and the IL-1 receptor accessory protein (IL-1RAcP), a common receptor subunit, which is involved also in IL-1 and IL-33 signaling [9]. Like IL-1, IL-18 or IL-33, IL-36 cytokines activate nuclear factor (NF)-κB, c-Jun N-terminal kinase (JNK) and extracellular signal-regulated kinase (ERK)-1/2 intra-cellular signaling pathways upon receptor binding [9]. IL-36Ra binds to IL-36R but does not induce any cellular response. It prevents the interaction of IL-36α, IL-36β and IL-36γ with IL-36R and thus, acts as a natural inhibitor [10].

IL-36R and its ligands are expressed in skin and internal epithelial tissues exposed to pathogens, such as trachea, lung and esophagus, but also in the brain, gut and kidney [5,11-13]. Several studies suggest that IL-36 exerts pro-inflammatory effects contributing to the pathogenesis of psoriasis and lung inflammation [11,14-16]. In addition, we recently described that IL-36 stimulates cytokine production by dendritic cells (DC) more efficiently than other IL-1 family members [17]. In addition, IL-36 acts in synergy with IL-12 to induce the polarization of naïve CD4^+ ^T cells into T helper (Th)1 cells [18]. Consistently, IL-36 enhances Th1 responses *in vivo *[17,18]. These observations led to the hypothesis that IL-36, being expressed in epithelia and in immune cells, might act as an early danger signal to activate cells of the innate and adaptive immune system. Depending on the context, this activation might enhance host responses against pathogens, or amplify pathological inflammation, as illustrated by the occurrence of generalized pustular psoriasis in patients with mutated IL-36Ra [19,20].

In a previous study, we examined the role of the IL-36 cytokines in human arthritis. IL-36α and IL-36β mRNA were detected in synovial biopsies of patients with rheumatoid arthritis (RA). Human synovial fibroblasts (hSF) and articular chondrocytes (hAC) expressed IL-36R and produced pro-inflammatory mediators, such as IL-6, IL-8 and nitric oxide (NO) in response to stimulation by recombinant IL-36β, but this effect was of a much lower magnitude than that induced by IL-1. In hSF, IL-36β mRNA levels were enhanced upon stimulation with IL-1β and/or TNF-α, while IL-36β mRNA expression was constitutive in hAC. IL-36β protein levels were detectable in the synovial fluid and in the serum of patients with RA. However, there was no correlation between serum levels of IL-36β and markers of the acute-phase response [21]. A recent study reported increased IL-36α protein expression in the synovial tissue of patients with RA and psoriatic arthritis (PsA), as compared to osteoarthritis (OA). In this work, IL-36α expression was mainly associated with CD138^+ ^plasma cells. IL-36R and IL-36Ra expression was similar in RA, PsA and OA synovium [22].

In the present study, we examined expression of IL-36 and IL-36R in joints of mice with collagen-induced arthritis (CIA), and investigated the role of IL-36R signaling in three different experimental models of arthritis.

## Materials and methods

### Mice

C57BL/6 mice were obtained from Janvier (Le Genest-St-Isle, France) or Charles River Laboratories (Wilmington, MA, USA) and were used between 9 and 13 weeks of age. DBA/1 mice were also obtained from Janvier and were used between 10 and 12 weeks of age. BALB/c mice were obtained from Jackson Laboratories (Bar Harbor, ME, USA) and were used between 8 and 10 weeks of age. IL-36R-deficient mice (IL-36R^-/-^) were backcrossed seven times into a pure C57BL/6J genetic background using a marker-assisted selection protocol (MASP) approach and were used between 8 and 12 weeks of age [23,24]. All mice were housed under conventional conditions, and water and standard laboratory chow were provided *ad libitum*. Animal studies were conducted under protocols approved by the Geneva Cantonal Authority for Animal Experiments (Geneva, Switzerland; licenses 31.1.1005/3351/2 and 31.1.1005/3834/2) or by the Institutional Animal Care and Use Committee at Amgen Inc. (Seattle, WA, USA).

### RNA extraction and determination of gene expression by quantitative real-time RT-PCR

Ankles were collected during the early phase of CIA (between days 22 and 30 after the first immunization) from paws with different severity of arthritis. Total RNA was extracted with Trizol reagent (Invitrogen AG, Basel, Switzerland) and reverse-transcribed using SuperScript II Reverse transcriptase (Invitrogen Life Technologies, Basel, Switzerland). The mRNA levels for genes of interest were examined by quantitative RT-PCR using the iQ SYBR Green Supermix (Bio-Rad, Hercules, CA, USA) according to a standard protocol (40 cycles, annealing temperature 60°C). All primer sequences are listed in Table 1. Relative levels of mRNA expression were normalized to glyceraldehyde-3-phosphate dehydrogenase (GAPDH) mRNA levels using a comparative method (2^-ΔCt^). Non-reverse-transcribed RNA samples and water were included as negative controls.

**Table 1:** Sequences of primers used for quantitative real-time polymerase chain reaction

IL-36R forward	5'-AAACACCTAGCAAAAGCCCAG-3'
IL-36R reverse	5'-AGACTGCCCGATTTTCCTATG-3'
IL-1R1 forward	5'- GAGTTACCCGAGGTCCAGTGG-3'
IL-1R1 reverse	5'-GAGGGCTCAGGATAACAGG-3'
IL-36γ forward	5'- AGAGTAACCCCAGTCAGCGTG-3'
IL-36γ reverse	5'- AGGGTGGTGGTACAAATCCAA-3'
IL-1β forward	5'-TGTGAAATGCCACCTTTTGA-3'
IL-1β reverse	5'-GTGCTCATGTCCTCATCCTG-3'
IL-36Ra forward	5'- CCTGCTTTCTACTTAGGTCTCAAAT-3'
IL-36Ra reverse	5'- GCTCCTCTGTCTCTCTATCCTCTAT-3'
IL-1Ra forward	5'- GGGATACTAACCAGAAGACC-3'
IL-1Ra reverse	5'- GACAGGCACAGCTTGCCCCC-3'
GAPDH forward	5'-AGGCCGAGAATGGGAAGCTTGT-3'
GAPDH reverse	5'-TACTCAGCACCGGCCTCACCC-3'

GAPDH, glyceraldehyde-3-phosphate dehydrogenase

### Antibodies and recombinant cytokine

The following monoclonal antibodies were produced by Amgen Inc.: a rat IgG2a anti-mouse IL-36R antibody (M616) and a murinized chimeric (hamster-mouse) IgG1 anti-mouse IL-1RI antibody (M147) directed against the extracellular domain of mouse IL-36R and IL-1RI, respectively; two rat IgG2a isotype control antibodies (4G8 and M10) and a mouse IgG1 isotype control antibody (4D2). We also used a rat IgG2a isotype control (9B5; anti-human CD44) antibody provided by Prof. Beat Imhof (University of Geneva School of Medicine, Geneva, Switzerland). Recombinant murine IL-36γ was produced at Amgen Inc. as an N-terminal truncated form displaying high biological activity [10].

### Assessment of *in vivo *blocking activity of the monoclonal anti-IL-36R antibody

The ability of the anti-mouse IL-36R antibody (M616) to inhibit the biologic effects of IL-36R agonists was tested *in vivo*. BALB/c mice were pre-treated intranasally (i.n.) with the anti-mouse IL-36R antibody (50 μg/mouse), an isotype- matched control antibody (M10; 50 μg/mouse) or PBS 2 h prior to i.n. challenge with recombinant murine IL-36γ (1 μg/mouse) on days 0, 1 and 2. Bronchoalveolar lavage (BAL) fluids recovered from mice 4 h after the last injection on day 2 were used to assess total and differential cell counts and to measure chemokine (CCL20, CCL11 and CCL24) protein levels.

### Bronchoalveolar lavage and differential cell counts

Mice were anesthetized with avertin (300 μL (5% solution)/mouse; Sigma-Aldrich; St Louis, MO, USA). BAL fluid was collected via endotracheal intubation using a 25 gavage needle with a 1 mm ball tip, by gently washing and aspirating 0.5 mL PBS solution twice. The 2 × 0.5 mL BAL fluid samples were pooled for each animal and centrifuged. The BAL fluid supernatant was immediately diluted 1:1 with a PBS, 0.1% Tween 20 solution. BAL sediments were re-suspended in PBS + 0.5% FBS and 175 μL per sample were used for counting with the ADVIA 120 Hematology system (Siemens Diagnostics, Tarrytown, NY, USA) to obtain an automated total of cell counts and differentials.

### Collagen-induced arthritis

CIA was induced in male DBA/1 mice, as previously described [25]. Blocking anti-IL-36R M616, anti-IL-1RI M147 antibodies or their respective isotype-matched control antibodies (4G8 and 4D2) were injected (150 μg/mouse intraperitoneally (i.p.)) every 2 to 3 days starting on day 27 after the first immunization. Mice were killed on day 41. During the course of CIA, arthritis severity was assessed by clinical scoring using a 3-point scale for each paw, as previously described [26].

### Antigen-induced arthritis (AIA)

Male C57BL/6 mice were immunized intradermally (i.d.) at the base of the tail with 100 μg of methylated BSA (mBSA; Fluka, Buchs, Switzerland), emulsified in complete Freund's adjuvant (Difco, Basel, Switzerland) containing 5 mg/mL *Mycobacterium tuberculosis*. On day 7, a booster injection of 100 μg mBSA in incomplete Freund's adjuvant (Difco) was given at the base of the tail. On day 21, arthritis was induced by intra-articular injection of 100 μg mBSA in 10 μL PBS into the left knee joint of mBSA-immunized mice, the right knee being injected with sterile PBS alone. The anti-IL-36R antibody M616 (150 μg/mouse, i.p.), the isotype-matched control antibody 9B5 (150 μg/mouse, i.p.) or PBS were injected 1 h before intra-articular mBSA injection. Mice were sacrificed 8 or 14 days after induction of arthritis, the latter group receiving a second injection of antibodies or PBS on day 4. The development of arthritis was followed by measuring ^99m^Technetium (Tc) uptake in the knees on days 1, 3 and 7 after intra-articular mBSA injection, as previously described [27]. A second AIA experiment was performed on adult male IL-36R^-/- ^and wild-type (WT) C57BL/6 mice. ^99m ^Tc uptake was measured in the knees on days 1, 3 and 7 after intra-articular mBSA injection and the mice were killed on day 8.

### K/BxN serum transfer-induced arthritis and clinical scoring

K/BxN serum was collected from 9-week old arthritic K/BxN mice, as previously described [28]. The serum samples were pooled and stored at -80°C until use. K/BxN serum transfer-induced arthritis was induced in adult female IL-36R^-/- ^and WT C57BL/6 mice by i.p. injection of K/BxN serum (200 μL) on days 0 and 2. Mice were scored clinically every day for the development of arthritis using a semi-quantitative scoring system [29]. The mice were sacrificed on day 6.

### Histological grading of arthritis

At sacrifice, isolated joints (knees for CIA and AIA; ankles for K/BxN serum transfer-induced arthritis) were fixed in 10% formalin, decalcified in 15% ethylenediaminetetraacetic acid (EDTA), and embedded in paraffin. Serial sections (3 μm) were stained with H&E for evaluation of inflammation or with toluidine blue to analyze cartilage damage. Sections were scored by a pathologist (CAS) in a blinded manner for arthritis severity by assessing inflammation and joint destruction with a semi-quantitative score, as described elsewhere [29].

### Measurement of cytokine and chemokine levels in serum, BAL fluids and tissue lysates

Serum, BAL fluids, and knee joints were collected at sacrifice. Total joint proteins were extracted as described previously [30]. Total protein concentrations were determined using the DC Protein Assay kit (Bio-Rad Laboratories, Hercules, CA, USA), according to the manufacturer's protocol. Chemokine (CCL20, CCL11, CCL24 and CXCL-1) and cytokine (IL-1Ra, IL-6) protein levels in serum, BAL and tissue lysates were determined using commercial Duoset ELISA Development Systems from R&D Systems (R&D Systems, Abingdon, UK).

### Statistical analysis

One-way analysis of variance (ANOVA) followed by two-tailed Student's *t*-test was used for statistical analysis of experiments involving more than two groups. Two-tailed Student's *t*-test was performed only when the one-way ANOVA yielded statically significant results. Differences in arthritis severity and in the number of affected paws at the end of the follow up were evaluated using the Kruskal-Wallis test. *P*-values < 0.05 were considered significant.

## Results

### Expression of IL-36R, IL-36γ and IL-36Ra in normal and inflamed joints during CIA

**Figure 1:**
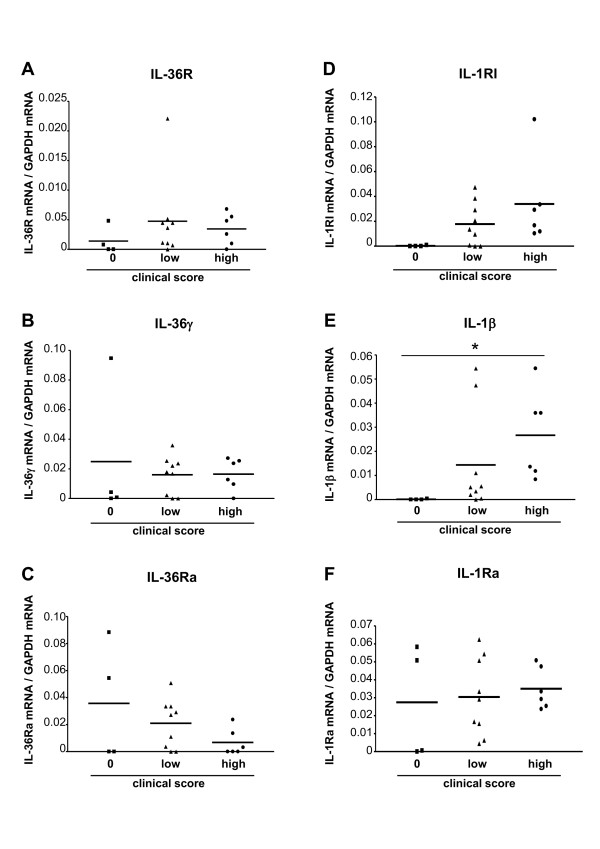
**Analysis of IL-36R, IL-36γ and IL-36Ra mRNA expression in normal and inflamed joints during collagen-induced arthritis (CIA)**. IL-36 receptor (IL-36 R) (**A**), IL-36γ (**B**), IL-36R antagonist (IL-36Ra) (**C**), IL-1RI (**D**), IL-1β (**E**) and IL-1Ra (**F**) mRNAs levels were determined by RT-qPCR in normal (clinical score = 0) or inflamed (low clinical scores: 0.5 to 1.5; high clinical scores: 2.0 to 3.0) ankles of type II collagen (CII)-immunized DBA-1 mice in the first nine days after the appearance of clinical symptoms (early arthritis). (**A-F**) mRNA expression was normalized to the amount of glyceraldehyde-3-phosphate dehydrogenase (GAPDH) mRNA. Each data point represents a single mouse; horizontal lines show the mean. *P < 0.05 versus score 0, as assessed by analysis of variance, followed by unpaired two-tailed Student's *t*-test.

We first examined expression of IL-36R and IL-36 ligands in the joints of mice with different severity of CIA using RT-quantitative (q)PCR. IL-36R, IL-36γ and IL-36Ra mRNA were expressed in clinically unaffected (clinical score = 0) and in inflamed joints (Figure 1A-C), whereas IL-36α and IL-36β mRNA were undetectable (data not shown). IL-36R, IL-36γ and IL-36Ra mRNA levels did not correlate with disease severity, but were comparable to those found in normal skin, a tissue in which IL-36 has been previously reported to play a pathogenic role (data not shown) [11]. IL-36R expression was constitutive in the joints of naïve mice, while IL-36γ and IL-36Ra expression levels were at least one order of magnitude lower than in joints of mice with CIA (data not shown). For comparison, and in agreement with previously published data, IL-1RI, IL-1β and IL-1Ra mRNAs were present in inflamed joints of mice with CIA (Figure 1D-F) with a significant correlation between IL-1β mRNA levels and the severity of arthritis [31].

### Efficacy of a blocking monoclonal anti-IL-36R antibody *in vivo*

**Figure 2:**
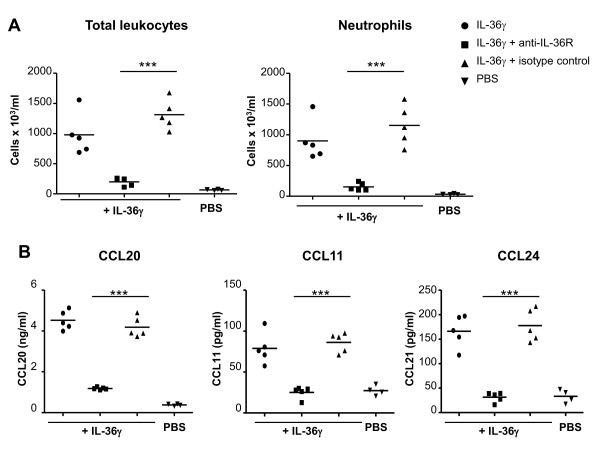
**Efficacy of a blocking monoclonal anti-IL-36 receptor (R) antibody *in vivo***. BALB/c mice (n = 5/group) were pre-treated intranasally (i.n.) with a rat IgG2a anti-mouse IL-36R antibody (M616; 50 μg/mouse; squares), an isotype control antibody (M10; 50 μg/mouse; triangles) or PBS (circles) 2 h prior to i.n. challenge with recombinant IL-36γ (1 μg/mouse) on days 0, 1 and 2. A fourth group of mice, used as negative control, only received i.n. injection of PBS (inverted triangles). On day 2, bronchoalveolar lavage fluids recovered from mice 4 h after the last injection were used to assess (**A**) total leucocyte and neutrophil counts and (**B**) chemokine (CCL20, CCL11 and CCL24) protein levels by DuoSet ELISA. (**A-B**) Results are shown as individual values for each mouse (symbols) and mean values (lines). ***P < 0.001 versus isotype control-treated mice, as assessed by unpaired two-tailed Student's *t*-test.

To investigate the role of IL-36R signaling in experimental arthritis, we used a blocking monoclonal rat anti-mouse IL-36R antibody (M616). The neutralizing efficacy of this antibody was first validated by examining its ability to inhibit the biological effects of recombinant (r) mouse IL-36γ *in vivo *(Figure 2). We treated mice intranasally with the anti-mouse IL-36R antibody, an isotype-matched control antibody or PBS prior to challenge with rIL-36γ. In agreement with previous data, we observed that i.n. instillation of rIL-36γ increased the total leucocyte numbers and resulted in marked neutrophilic infiltration in BAL fluids (Figure 2A) [15]. The administration of rIL-36γ also increased the protein levels of several chemokines in BAL fluids, including CCL20 and CCL11, as previously described, as well as CCL24 (Figure 2B) and CXCL-1 (data not shown) [15]. Pre-treatment of mice with the anti-mouse IL-36R antibody inhibited the effects of rIL-36γ (Figure 2), indicating that this monoclonal anti-IL-36R antibody efficiently inhibits IL-36R signaling *in vivo*.

### Treatment with a monoclonal anti-IL-36R antibody does not modify the course of CIA

**Figure 3:**
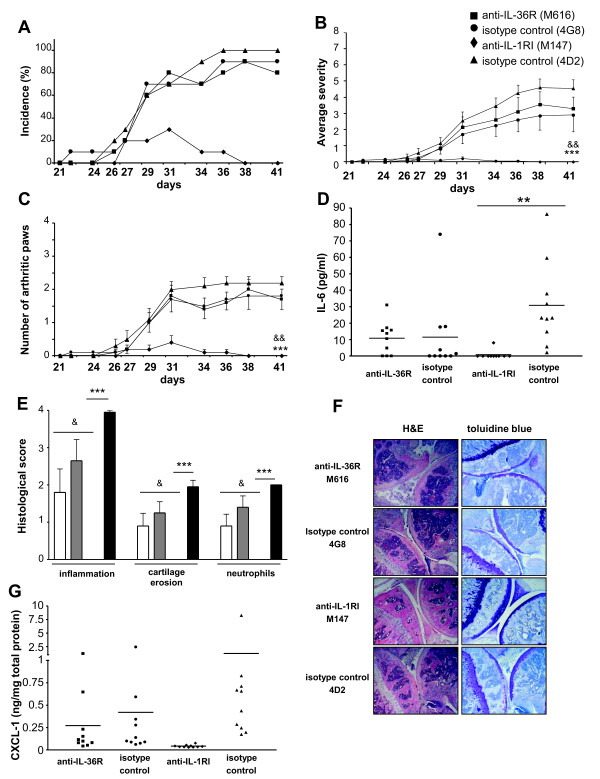
**Treatment with a monoclonal anti-IL-36 receptor (R) antibody does not modify the course of collagen-induced arthritis (CIA)**. CII-immunized mice (n = 10/group) were treated with anti-IL36R (M616, squares, white columns), anti-IL-1RI (M147, triangles, dashed columns) or isotype-matched control antibodies (4G8 (circles, grey columns) for M616, 4D2 (triangles, black columns) for M147), as described in Materials and methods. Results show the incidence of arthritis (**A**), the clinical severity of articular inflammation (**B**) and the number of arthritic paws (**C**) on days 21 to 41. The systemic inflammatory response (**D**) was assessed by measuring circulating IL-6 levels on day 41 after the first immunization. Joint sections from all mice were evaluated on day 41 for histological scores (**E**) and histological features (**F**). All sections were scored for inflammation, cartilage erosion and neutrophil infiltration. Images are representative of H&E- or toluidine blue-stained sections of knee joints for each group (original magnification × 10). (**G**) Levels of CXCL-1 (ng/mL) were determined by ELISA in ankle extracts of each mouse on day 41 and normalized by the total protein concentration (mg/mL). (**B, C, E**) Values are the mean ± standard error of the mean. ***P < 0.001, anti-IL-1RI versus isotype control 4D2; ^&^*P *< 0.05 and ^&&^*P *< 0.01, anti-IL-1RI versus anti-IL-36R, as assessed by Kruskal-Wallis test (**B **and **C**) or by analysis of variance (ANOVA), followed by unpaired two-tailed Student's *t*-test (**E**). (**D, G**) Results are shown as individual values for each mouse (symbols) and mean values (lines) **P < 0.01, anti-IL-1RI versus isotype control 4D2, as assessed by ANOVA, followed by unpaired two-tailed Student's *t*-test.

Consecutively, we tested the role of IL-36R signaling in CIA using DBA/1 mice. We compared the effect of an i.p. injected anti-IL-36R antibody, administered at disease onset, with that of an isotype-matched control antibody. An additional group of DBA/1 mice served a positive control and was treated with a blocking anti-IL-1RI antibody to compare the relative contributions of IL-36R ligands and IL-1 cytokines (IL-1α and IL-1β) to the development and severity of CIA. Treatment of mice with the blocking anti-IL-36R antibody was devoid of effect on the incidence (Figure 3A) or severity of CIA (Figure 3B and 3C). In contrast, inhibition of IL-1RI signaling resulted in marked attenuation of the disease. Consistent with these results, histological examination at the end of the experiment showed a complete protection in mice treated with the anti-IL-1RI antibody, whereas articular inflammation and structural damage were similar in mice injected with the anti-IL-36R or with isotype-matched control antibodies (Figure 3E and 3F). Similarly, serum IL-6 levels were elevated in mice treated with the anti-IL-36R antibody or with isotype-matched control antibodies, while circulating IL-6 was undetectable in anti-IL-1RI antibody-treated mice (Figure 3D). Finally, the levels of different inflammatory mediators, such as IL-6 and CXCL-1, were elevated in the joints of anti-IL-36R antibody and isotype control antibody-treated mice, while they were drastically decreased in mice treated with the anti-IL-1RI antibody (Figure 3G; data not shown). Taken together, our results suggest that there is no major contribution of IL-36R ligands to the development and severity of CIA.

### The course of AIA is not modified by treatment of wild-type mice with a monoclonal anti-IL-36R antibody or in IL-36R-deficient mice

**Figure 4:**
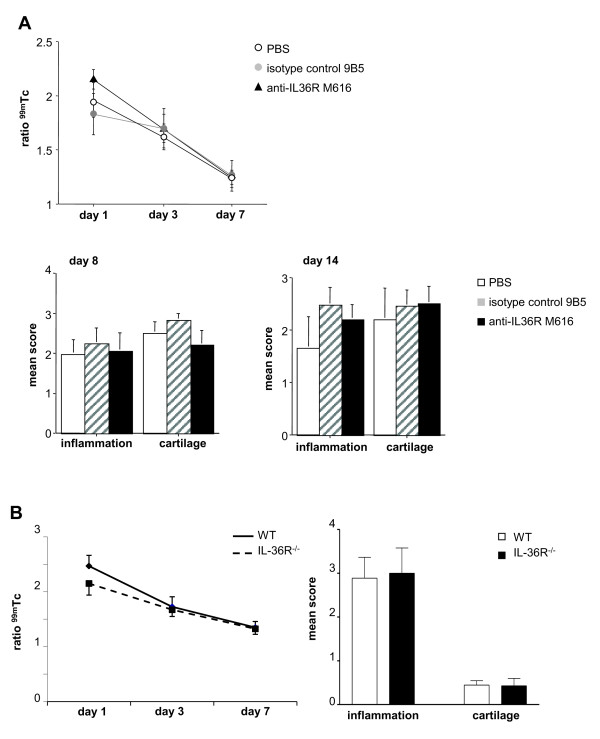
**The course of antigen-induced arthritis (AIA) is not modified by treatment with a monoclonal anti-IL-36 receptor (R) antibody or in IL-36R-deficient mice**. Time course of knee joint inflammation is shown in anti-IL-36R antibody-treated mice (**A**, upper panel) and in IL-36R knockout (KO) mice (**B**, left panel) with AIA. Joint inflammation was measured by external gamma counting of ^99m^technetium (Tc) accumulation on days 1, 3 and 7 after mBSA injection into the left knee. Results are expressed as the ratio of ^99m^Tc uptake in the left arthritic knee over the right non-inflamed knee. For each time point, the mean ± standard error of the mean (SEM) of the ratios is shown for anti-IL-36R antibody (n = 7; triangles), 9B5 (rat IgG2a anti-human CD44) isotype-matched control antibody-treated mice (n = 7; gray circles), or PBS-treated mice (n = 11; open circles), wild-type (WT) C57BL/6J mice (n = 9; black line) and IL-36R^-/- ^mice (n = 7; dashed line). (**A**, lower panels; **B**, right panel) Histological scores for synovial inflammation and cartilage degradation 8 and 14 days after intra-articular mBSA injection. Results shown represent the mean ± SEM for anti-IL-36R antibody (n = 5 on day 8, n = 6 on day 14, black columns), 9B5 isotype control (n = 3 on day 8, n = 4 on day 14, hatched columns), PBS-treated mice (n = 3 on day 8, n = 4 on day 14, open columns) (**A**, lower panels), WT C57BL/6J mice (n = 9 on day 8, white columns) and IL-36R^-/- ^mice (n = 7 on day 8, black columns) (**B**, right panel).

To extend our investigations to another model of arthritis and mouse strain, we then analyzed the effect of the anti-IL-36R antibody (M616) on the course of AIA in C57BL/6 mice. The severity of AIA, as assessed by ^99m^Tc uptake, was not altered by the treatment with the anti-IL-36R antibody, initiated at the time of disease induction, as compared to treatment with an isotype-matched control antibody or with PBS (Figure 4A, upper panel). Accordingly, histological analysis of the paws on days 8 and 14 of arthritis showed no difference in the extent of articular inflammation and structural damage between mice treated with the anti-IL-36R antibody and the control groups (Figure 4A, lower panel). To further examine the role of IL-36R signaling in the control of joint inflammation in this model, AIA was induced in IL-36R^-/- ^and WT control mice. As shown in Figure 4B, the severity of arthritis, assessed by Tc uptake and histological analysis did not differ between IL-36R^-/- ^and WT mice. Taken together, these data suggest that the IL-36/IL-36R system is not of functional importance in AIA.

### Incidence and severity of K/BxN serum transfer-induced arthritis are not altered in IL-36R-deficient mice

**Figure 5:**
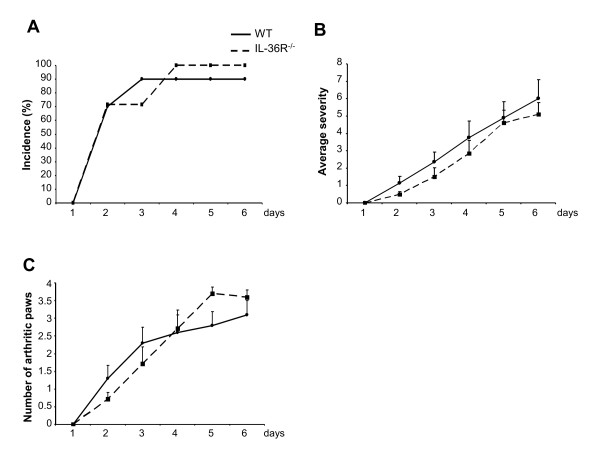
**Incidence and severity of K/BxN serum transfer-induced arthritis are not reduced in IL-36 receptor (R) knockout (KO) mice**. Incidence of arthritis (**A**), arthritis severity scores (**B**) and the number of affected paws (**C**) are shown for WT (n = 10, black line) and IL-36R KO (n = 7, dashed line) mice. (**A **and **C**) Results are shown as the mean ± standard error of the mean.

CIA and AIA are dependent on active immunization and are therefore influenced by alterations of the adaptive immune response. To more specifically investigate the involvement of IL-36R in the inflammatory effector phase of arthritis, we used the passive K/BxN serum transfer-induced model, which is independent of the adaptive immune response in IL-36R^-/- ^and WT control mice. As shown in Figure 5, we observed no difference in the incidence and the severity of the disease between IL-36R^-/- ^and wild-type mice.

## Discussion

This is the first study that examined in detail the expression of IL-36 cytokines and their receptor, as well as the role of IL-36R signaling in experimental arthritis. IL-36R, IL-36γ, and IL-36Ra mRNAs were expressed in the joints of mice with CIA but, as opposed to IL-1β, their levels did not correlate with the severity of articular inflammation. In addition, the development and severity of CIA was markedly attenuated by the administration of a blocking anti-IL-1RI antibody, but not by a neutralizing anti-IL-36R antibody. Similarly, anti-IL-36R antibody treatment did not modify disease severity in AIA. Finally, data obtained in IL-36R-deficient mice confirmed that the severity of arthritis was independent of IL-36R signaling in AIA and K/BxN serum transfer-induced arthritis, two experimental models that are dependent and independent of adaptive immune responses, respectively.

The role of adaptive immunity has been extensively examined in CIA, in particular that of the cytokines produced by the different CD4+ Th cell subsets. The pathogenic role of Th17 cells has been well demonstrated by using neutralizing antibodies and *il17a *gene knockout mice [32,33]. In contrast, Th1 responses exert more complex effects during the course of CIA. Indeed, IFN-γ^-/- ^and IFN-γ R^-/- ^mice exhibit a more severe form of CIA than WT mice, most likely due to the absence of a counter-regulatory effect of IFN-γ on the expansion of Th17 cells [34-36]. However, the injection of recombinant IL-12 enhances the severity of CIA during the induction phase, but attenuates the inflammatory process when administered in established CIA. Consistent with these findings, opposite phenotypes were observed in mice injected with neutralizing anti-IL-12 antibodies [37]. These data are in line with the early development of antigen-specific IFN-γ-producing T cells before the occurrence of overt signs of arthritis, whereas IL-17 production seems to increase later in the course of CIA [30]. We have recently shown that IL-36 induces the production of several cytokines and chemokines by bone marrow-derived DCs, and in particular the production of IL-12 [17]. Furthermore, IL-36 stimulates the polarization of naïve mouse Th0 cells into IFN-γ-producing Th1 cells, and this effect is dependent on the presence of IL-12 *in vitro *and *in vivo*. Of note, in the same conditions IL-1 had no effect on IFN-γ production [18]. In contrast, IL-1 has previously been shown to markedly stimulate the polarization of Th17 cells [38-40]. In agreement with these findings, excessive IL-1 signaling in IL-1Ra-deficient BALB/c mice is associated with the development of polyarthritis related to the enhanced production of IL-17 [41]. In addition, conditional myeloid-specific IL-1Ra-deficient mice had a more severe form of CIA with increased IL-17 levels in the joints and in cultured lymph node cells [30]. Taken together, these findings suggest that IL-36 and IL-1 exert distinct effects on Th cell responses that may, at least partly, explain the differences observed in the development and severity of CIA upon targeting IL-1RI or IL-36R. In addition, as illustrated also by our results in the K/BxN serum transfer-induced arthritis model, IL-1 and IL-36 may also exert distinct effects in the control of local inflammatory responses in the joint.

The injection of a neutralizing anti-IL-36R antibody at the time of arthritis induction did not modify the course of disease in AIA. Furthermore, the severity of AIA was not decreased in IL-36R^-/- ^mice, indicating that IL-36R signaling was also dispensable during the induction of the immune response against mBSA. This result is consistent with our previous data demonstrating normal IFN-γ production by antigen-specific T cells after immunization of IL-36R^-/- ^mice with Freund's adjuvant, but contrasts with the adjuvant effect of IL-36, as well as with the decreased IFN-γ production observed in IL-36R^-/- ^mice after infection with *Bacillus Calmette-Guérin *(BCG) [17,18]. The explanation for these differences may lie in the very potent effect of the complete Freund's adjuvant, which is able to overcome the absence of IL-36R signaling by stimulating a number of other cytokines involved in Th1 polarization, such as IL-12 and IL-18.

Finally, the severity of K/BxN serum transfer-induced arthritis was not affected by IL-36R deficiency indicating that the inflammatory effector phase of arthritis in this model was also independent of IL-36. In contrast, IL-1RI signaling is critically needed for the development of K/BxN serum transfer-induced arthritis [42]. Accordingly, we recently observed that administration of the anti-IL-1RI antibody markedly decreased the severity of serum transfer-induced arthritis (G. Palmer *et al*, unpublished data). These findings point to marked differences between IL-1 and IL-36 in the magnitude of their stimulatory effects on the production of pro-inflammatory mediators by human synovial fibroblasts and articular chondrocytes [21].

In conclusion, our results indicate, that as opposed to IL-1RI activation, IL-36R signaling is not involved in the development of experimental arthritis in the three models examined. The role of IL-1 in Th17 differentiation, as well as its potent stimulatory effects on resident joint cells leading to local inflammation, neither of which are shared by IL-36, may explain the differential involvement of these two cytokines in arthritis.

## Conclusions

Taken together with our previously published data on IL-36 levels in synovial tissue and fluid samples from RA patients, the results obtained here using three different models of experimental arthritis suggest that the IL-36/IL-36R pathway is unlikely to be involved in the pathogenesis of RA.

## Abbreviations

AIA: antigen-induced arthritis; ANOVA: analysis of variance; BAL: bronchoalveolar lavage; BSA: bovine serum albumin; CIA: collagen-induced arthritis; EDTA: ethylenediaminetetraacetic acid; ERK: extracellular signal-regulated kinase; FBS: fetal bovine serum; GAPDH: glyceraldehyde-3-phosphate dehydrogenase; hAC: articular chondrocytes; H&E: hematoxylin and eosin; hSF: human synovial fibroblasts; i.d.: intradermal; IFN: interferon; IL: interleukin; IL-36R: interleukin 36 receptor; IL-36R^-/-^: IL-36R-deficient mice; IL-1RAcP: IL-1 receptor accessory protein; IL-1Ra: IL-1 receptor antagonist; i.n.: intranasal; i.p.: intraperitoneal; JNK: c-Jun N-terminal kinase; KO: knockout; MASP: marker-assisted selection protocol; NO: nitric oxide; NF-κB: nuclear factor-κB; OA: osteoarthritis; PBS: phosphate-buffered saline; PsA: psoriatic arthritis; RA: rheumatoid arthritis; RT-PCR: reverse-transcriptase polymerase chain reaction; technetium: Tc; Th: T helper; TNF: tumor necrosis factor; WT: wild-type.

## Competing interests

CAS is an employee of Novartis Pharma AG, Basel, Switzerland. JET is an employee of Amgen Inc., Seattle, WA, USA. The other authors declare no financial or commercial conflict of interest.

## Authors' contributions

CL and GP planned studies, performed experiments, analyzed data and wrote the manuscript. ER, PM, SV, DTA and JET did experiments and analyzed data. CAS performed the histological scoring, analyzed data and wrote the manuscript. CG supervised the project, planned studies, analyzed data and wrote the manuscript. All authors have been involved in drafting the manuscript or in revising it critically for important intellectual content, and have read and approved the final manuscript.
